# Improving Pediatric Emergency Department Lethal Means Safety Documentation Rates for Adolescents with Mental Health Concerns

**DOI:** 10.1097/pq9.0000000000000903

**Published:** 2026-07-29

**Authors:** Cassandra L. Stegall, Ana-Maria McGill, John Fanton, Paul C. Mullan

**Affiliations:** From the * Department of Pediatric Emergency Medicine, Children’s Hospital of Philadelphia, Philadelphia, Pa.; † Department of Psychiatry, Eastern Virginia Medical School at Old Dominion University, Children’s Hospital of the King’s Daughters, Norfolk, Va.; ‡ Department of Psychiatry, Children’s Hospital of the King’s Daughters, Norfolk, Va.; § Department of Pediatric Emergency Medicine, Children’s Hospital of the King’s Daughters, Norfolk, Va.

## Abstract

**Introduction:**

Lethal means safety counseling (LMSC) is recommended for children presenting to the emergency department (ED) for a mental health (MH) evaluation. Currently, no gold-standard guidelines exist for documenting LMSC in the ED.

**Methods:**

This quality improvement project was conducted in the ED of a tertiary children’s hospital. Baseline data were analyzed for access to any LMSC documentation (ALD) for “firearms/guns,” “sharps,” or “medication,” and for complete LMSC documentation (CLD) if access to all 3 types was documented. The first intervention period added open-ended screening questions about access to lethal means to the MH-licensed clinical social worker note template. Intervention period 2 implemented an electronic health record “PowerForm” in Cerner PowerChart. We aimed to increase ALD and CLD to exceed 75% and 50%, respectively, during a 6-month period, with sustained success. Statistical process control p-charts with standardized special-cause rules were used to determine performance over time.

**Results:**

Baseline ALD and CLD rates were 3% and 1%, respectively. During intervention 1, the ALD and CLD rates increased to 81% and 68%, respectively, with special-cause variation noted for both rates. During intervention 2, ALD increased to 90%, but CLD rates did not change significantly (66%). For intervention 2, the documentation of firearm/gun access (90%) was higher than documentation of access to sharps (68%) or medication (69%).

**Conclusions:**

We achieved a significant increase in ALD and CLD for MH evaluations in our ED. Lessons learned may generalize to other ED settings.

## INTRODUCTION

Suicide remains the second leading cause of adolescent death, with rates of 2.3 per 100,000 persons for 10- to 14-year-olds and 13.5 per 100,000 persons for 15- to 24-year-olds.^1^ Rates among adolescents have increased at a mean annual rate of 9.2% since 2016.^2^ Firearms are the most common means for suicide completion, with a lethality rate of 90%.^3–5^ Adolescents living in homes with unsafely secured firearms are more likely to complete suicide using firearms.^6,7^ The risk of suicide mortality triples if a gun is stored loaded and independently doubles if it is stored unlocked.^8^ Intentional medication ingestions are the most common means of suicide attempt but have lower lethality compared with firearms.^1,9,10^ Self-harm causes 35,004 injuries a year among adolescents aged 10–14 years.^1^

Lethal means safety counseling (LMSC) is an effective suicide prevention strategy to decrease access to commonly used lethal means.^11,12^ Stricter firearm regulation is associated with a small reduction in firearm-specific suicide deaths.^10^ It is recommended that all clinicians and trainees receive LMSC education as part of medical education.^13^ The American Academy of Pediatrics (AAP) and the Joint Commission recommend that LMSC be done at all emergency department (ED) mental health visits due to the high ED use rates by those at highest risk for suicide.^13–15^ The AAP recommends that LMSC include the removal of lethal means, including medicines, toxic substances, sharp objects, and firearms, and be given to individuals at risk of harming themselves or others.^13^ Studies demonstrate that for patients seen in the ED for a mental health complaint, LMSC and safe storage interventions are effective in enacting safe firearm storage practices.^16,17^

This study aimed to increase LMSC documentation in our pediatric ED for patients seen for mental health concerns. It expands upon current literature by including medications and sharps/knives in LMSC, as prior studies have focused on firearms.^18–21^ This study also proposes a PowerForm template to document and encourage screening and counseling for other institutions, given the varying documentation language in the literature. Our primary aims were to increase any LMSC documentation (ALD) to greater than 75% in 6 months and to increase complete LMSC documentation (CLD) to greater than 50% in 6 months.

## METHODS

### Context

This project is a quality improvement initiative within a freestanding children’s hospital ED that sees 60,000 patients a year. During the study period, the number of mental health evaluations was 2,671 in 2023, 3,032 in 2024, and 3,021 in 2025. The ED includes a 6-bed mental health emergency services treatment area adjacent to the ED and staffed by the ED team. This institution opened an inpatient psychiatric facility in October 2022, which was associated with increases in annual admissions from 401 in 2023 to 782 in 2024 and 709 in 2025. Additionally, the median ED length of stay for admitted patients decreased from 17.5 hours in 2023 to 10 hours in 2024 and to 8 hours in 2025.

All ED patients 10 years and older are screened for suicide with the Ask Suicide Screening Questionnaire.^22^ Patients with a positive Ask Suicide Screening Questionnaire are triaged and assessed, with dispositions developed based on their immediate medical clearance, acuity, history, and safety assessments. All ED patients with a mental health chief complaint receive a mental health evaluation by a licensed clinical social worker (SW). This institution uses Cerner as its electronic medical record (EMR).

An internal funding grant of $5,000 was used to provide cable gun locks and medicine lockboxes for families. Cable gun locks were available in the ED waiting room and for SW dispersal. Before this project, a working group, including pediatric emergency physicians, psychiatrists, SWs, and SW management, met to discuss current LMSC documentation, project buy-in, and anticipated barriers. The project baseline period ran from April 1, 2023, to July 31, 2023.

### Subjects

Inclusion criteria included patients seen between 12 and 18 years of age who presented with a primary mental health concern, received a mental health evaluation during their ED visit by a licensed clinical SW, and were discharged from the ED. The age of 12 was chosen based on a prior AAP multisite quality improvement project, Improving Mental Health Processes, Workflows, and Responses, which used age 12. Exclusion criteria included external transfers, admitted patients, and those who left before treatment was completed (including against medical advice departures, elopements, and patients who left without being seen).

### Interventions

**Fig. 1.**
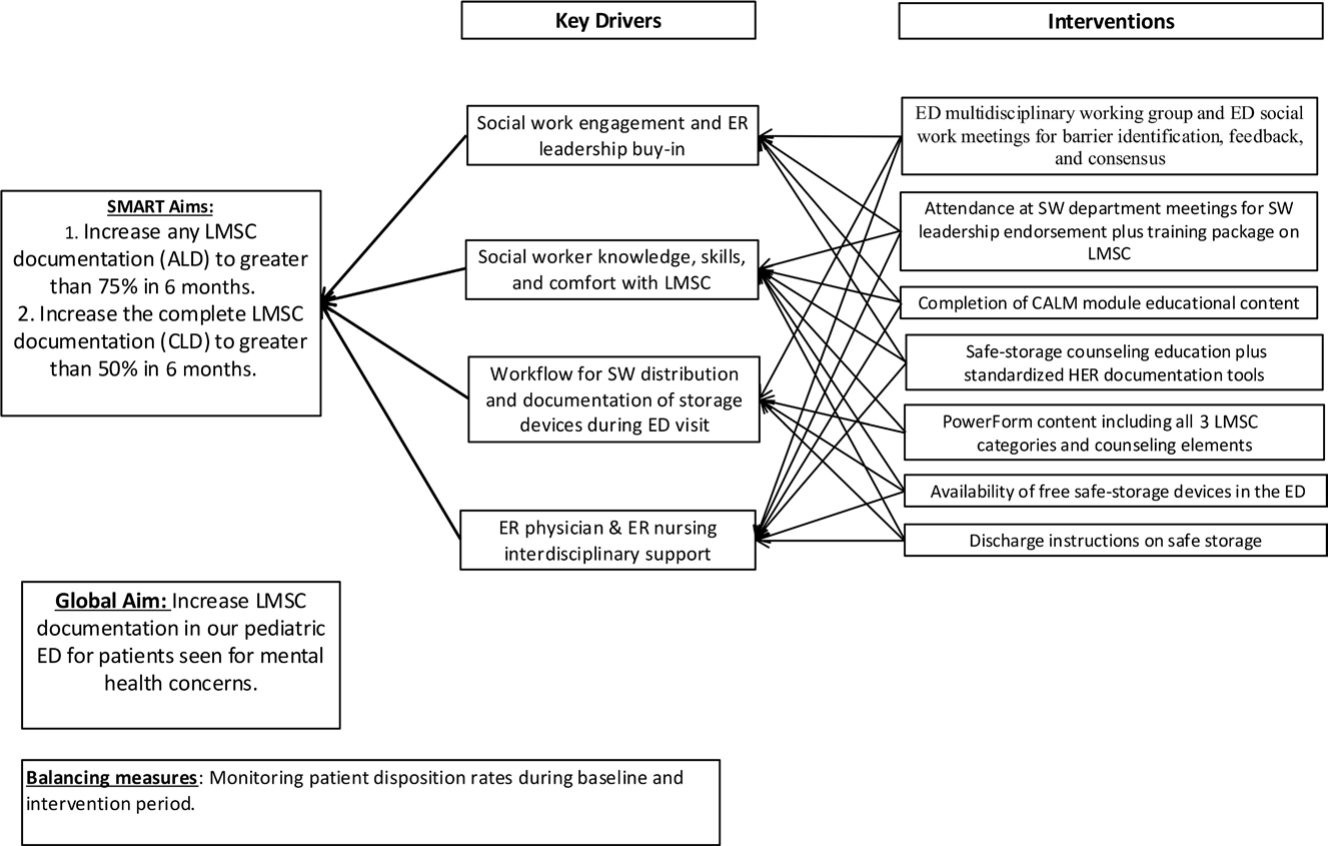
Key driver diagram demonstrating key drivers and interventions to meet the SMART and global aims.

After identifying stakeholders, a multidisciplinary workgroup met to identify primary drivers and interventions (Fig. 1). The primary drivers included ED SW, ED leadership stakeholder buy-in, knowledge, skills, and comfort with LMSC, SW workflow for distribution and documentation of storage devices, and ED physician and nursing support. Secondary drivers for change identified during intervention periods 1 and 2 included SW note template screening questions, EMR PowerForm implementation, attending ED SW department meetings for leadership endorsement, creating and distributing training presentations with the completion of the counseling on access to lethal means module,^9^ having free cable gun locks and medicine lockboxes, and finally, discharge instructions that contained educational counseling on safe storage (Fig. 1). SW perceptions and feedback were incorporated during the intervention period through distribution of a survey.^23^

### Intervention Period 1

**Fig. 2.**
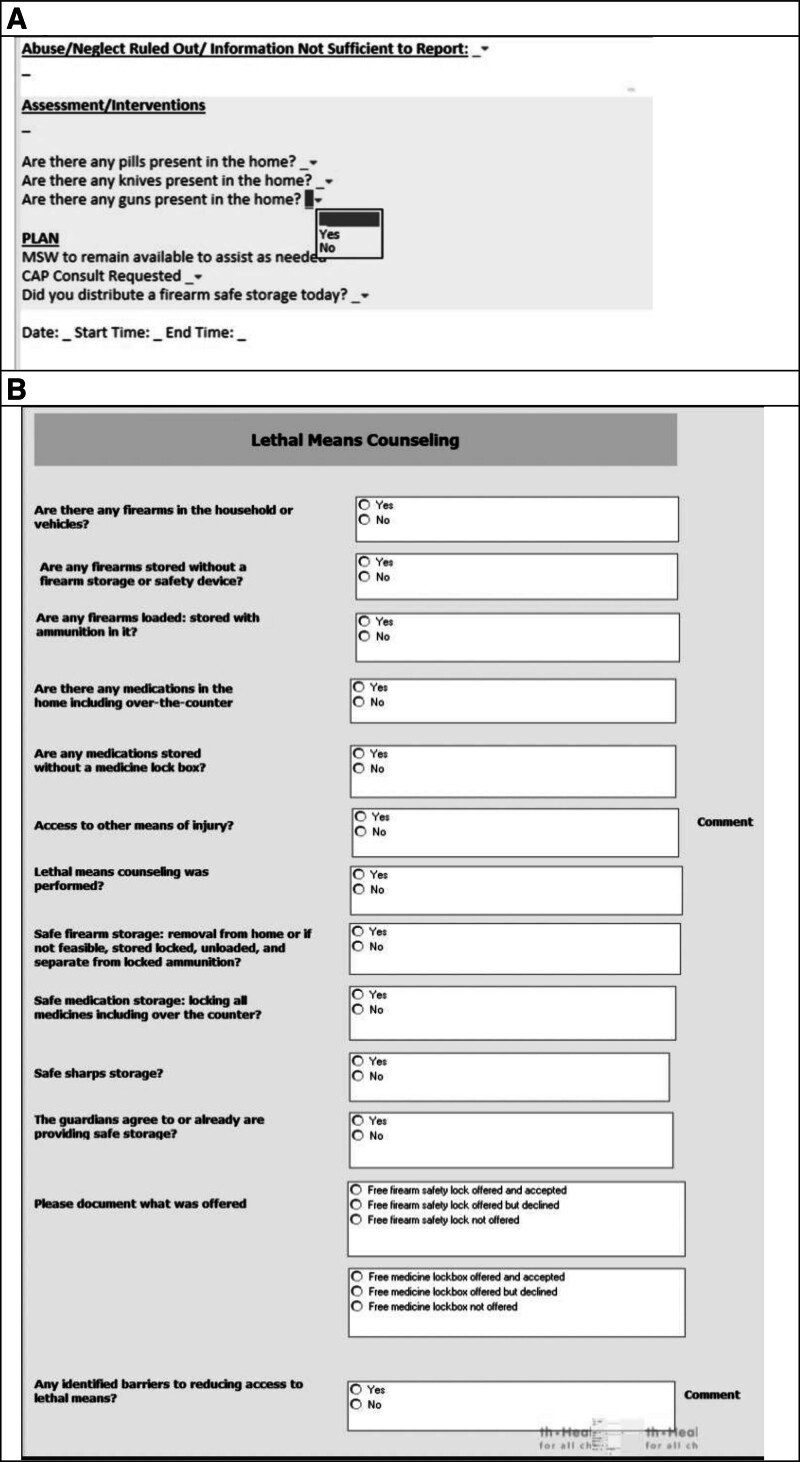
LMSC documentation templates. A, Documentation of the LMSC screening questions implemented in August 2023. B, Documentation of the PowerForm implemented in August 2024.

Our first intervention period began on August 1, 2023, and included the introduction of screening questions about access to 3 types of lethal means into the SW note template (Fig. 2). Questions were added as standard drop-down options, with a free-text response area. Questions were optional because the EMR form lacked mandatory response options. Other activities during intervention 1 included providing free cable firearm locks in our ED, in conjunction with an educational PowerPoint presentation sent to all SWs for review, and encouraging SWs to complete the counseling on access to lethal means module to enhance their knowledge of how to conduct LMSC.^9^ One of the authors attended the ED’s monthly social work department meetings during the first few months of intervention 1 to answer questions and encourage project support.

### Intervention Period 2

Intervention 2 started on August 1, 2024, with the implementation of a new EMR PowerForm for ED SWs to complete (Fig. 2). A PowerForm is a digital clinical document template within the Cerner EMR that collects and organizes data via data-entry fields and conditional logic.^24^ An expert working group of ED clinicians and SWs met to design the form, incorporating language from the published literature on documenting LMSC.^18,25^ They added questions that included addressing counseling measures, firearm triple safe storage, and the presence of firearms in a family vehicle. One question also documented whether the ED SW had distributed cable gun locks or medicine lockboxes during the ED visit.

### Study of the Interventions

A random retrospective sample of 20 monthly charts of included patients was reviewed during the baseline and intervention periods. Systematic random sampling was used, with a sampling interval of 5 for charts that met the inclusion criteria and a random start. Sampling continued until the goal of 20 charts was met. The SW evaluation notes and the PowerForm were searched using keywords related to LMSC, such as “firearm,” “gun,” “medication,” “medicine,” “sharp,” and “knife.”

### Measures

Our LMSC documentation outcome measures were to determine the extent of ALD and CLD. For ALD, we considered “any” documentation as sufficient if any of the 3 lethal means were documented in the EMR: firearms/guns, sharps/knives, or medications. For CLD, all 3 lethal means needed to be documented.

### Analysis

**Fig. 3.**
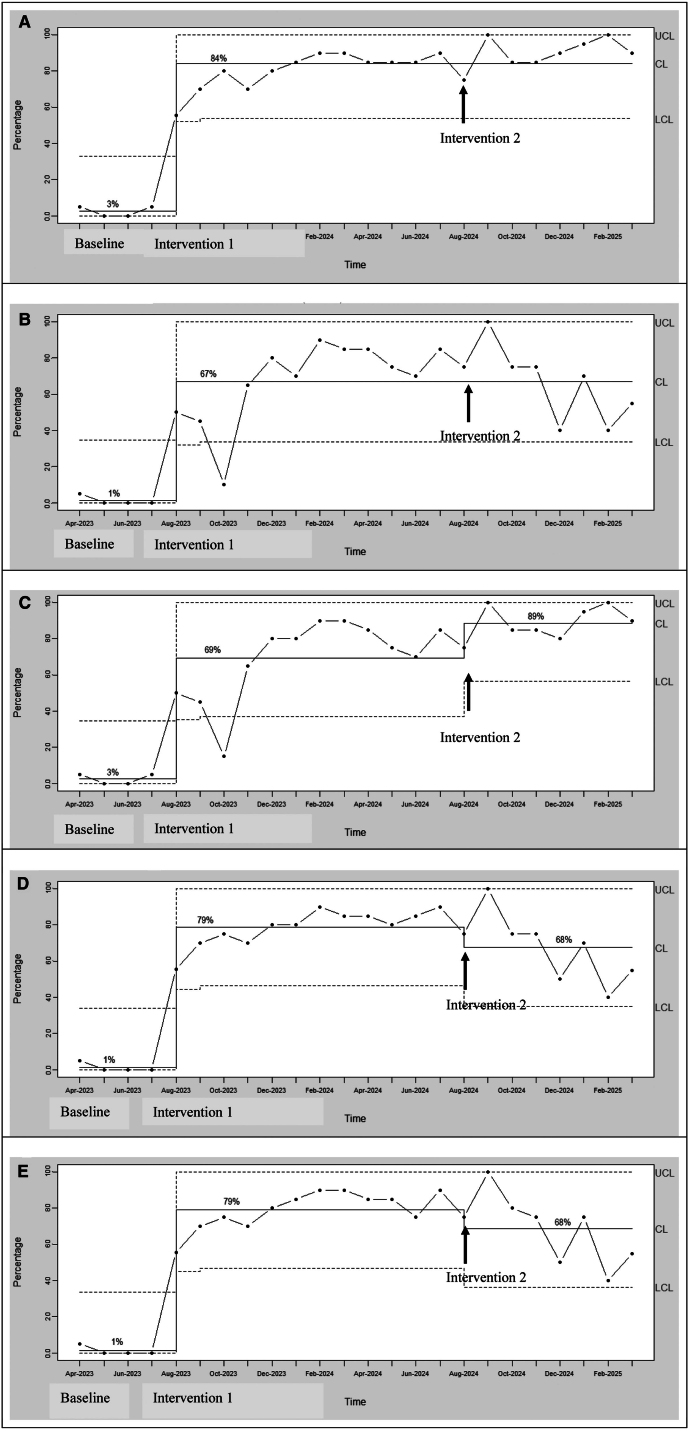
Statistical process control charts of LMSC documentation during the baseline (April 2023–July 2023), intervention period 1 (August 2023–July 2024), and intervention period 2 (August 2024–March 2025). A, ALD is any lethal means documentation: firearms, medicines, or knives. B, CLD is complete lethal means documentation: firearms, medicines, and knives. C, Firearm access documentation. D, Knives/sharps access documentation. E, Medication access documentation. CL, center line; LCL, lower control limit; UCL, upper control limit.

Documentation rates were measured over time through statistical process control p-charts. Established criteria were applied to assess special-cause variation in LMSC documentation during the intervention periods.^26^ In Figure 3, the *y* axis shows the percentage of visits with documentation, and the *x* axis shows time. The solid line is the centerline, with dashed lines indicating the upper and lower control limits. The χ^2^ test was used to compare the proportion of discharges between the baseline and intervention phases, as well as differences in proportions for documentation of firearms compared with sharps/knives and medications. Table 1 presents a descriptive summary of our results, with the numerator representing the number of documented cases and the denominator representing the number of patient encounters that met the inclusion criteria.

**Table 1.** Descriptive Summary of LMSC Documentation Proportions across the Study Periods

	Baseline (Numerator/Denominator)*	Intervention 1 (Numerator/Denominator)*	Intervention 2 (Numerator/Denominator)*
ALD	2.5% (2/79)	80.7% (192/238)†	90.0% (144/160)†
CLD	1.3% (1/79)	67.6% (161/238)†	66.3% (106/160)
Firearms documentation	2.5% (2/79)	69.3% (165/238)†	88.8% (142/160)†
Sharps/knives documentation	1.3% (1/79)	78.6% (187/238)†	67.5% (108/160)†
Medication documentation	1.3% (1/79)	79.0% (188/238)†	68.8% (110/160)†

* Numerator represents the number of documented cases, and the denominator represents the number of patient encounters that met the inclusion criteria.

† *0* < 0.05 for this period versus the prior period.

### Ethical Considerations

The Eastern Virginia Medical School institutional review board approved this study as exempt research because it was a quality improvement initiative and classified it as not involving human subjects research.

## RESULTS

### Differences in Baseline and Intervention Periods

Based on our inclusion criteria, 79 charts were reviewed during the baseline period, 238 during intervention 1, and 160 during intervention 2. Of the charts meeting exclusion criteria, there were 269 in the baseline period, 928 in intervention 1, and 124 in intervention 2; July 2023 (baseline) and August 2023 (intervention period 1) did not meet the goal of 20 charts per month, likely due to lower patient volumes. There was no difference in the proportion of discharges between the baseline and intervention 1. A small but statistically significant decrease in the proportion of included patients was observed in intervention 2 compared with intervention 1 (*P* < 0.05) (Table 1). Of note during the study period, the median ED length of stay for admitted patients decreased from 17.5 hours in 2023 to 10 hours in 2024 and to 8 hours in 2025.

### Any LMSC Documentation

ALD rates increased from 2.5% at baseline to 81% and 90% in interventions 1 and 2, respectively. In intervention 1, special-cause variation was met in August 2023 with 1 point outside of 3-sigma limits. In intervention 2, following the implementation of the PowerForm, there was a 7-point run in March 2025 (Fig. 3).

### Complete LMSC Documentation

CLD rates increased from 1% in the baseline to 68% and 66% in interventions 1 and 2, respectively. In intervention 1, special-cause variation was observed in August 2023, with 1 point outside the 3-sigma limits. However, in intervention 2, special-cause criteria were met for CLD rates in the wrong direction, with 2 of 3 points between −2 and −3 sigma in February of 2025 (Fig. 3).

### Types of Lethal Means Documentation

Firearm lethal means documentation increased from 3% at baseline to 70% and 89% in interventions 1 and 2, respectively. In intervention 1, special-cause variation was observed in August 2023, with 1 point outside the 3-sigma limits, and in June 2024, with 8 or more points above the centerline. During intervention 2, the proportion of firearms documented was significantly higher than sharps/knives (difference 21.3; 95% confidence interval, 11.9–30.6; *P* < 0.001) and medications (difference 20; 95% confidence interval, 10.7–29.3; *P* < 0.001) (Fig. 3).

Sharps/knives rates increased from 1% at baseline to 79% and 68% in interventions 1 and 2, respectively. Similarly, medication rates increased from 1% at baseline to 79% and 69% in interventions 1 and 2, respectively. For both sharps/knives and medications in intervention 1, special-cause variation was observed in August 2023, with 1 point outside the 3-sigma limits. In intervention 2, special-cause variation was observed in February 2025, with all 3 points below the 2-sigma limit, indicating a decrease in safe sharps and medication documentation (Fig. 3).

## DISCUSSION

This project met its primary aim to increase the ALD rate to greater than 75% and the CLD rate to greater than 50% within 6 months. This was accomplished through 2 intervention periods, which included the key drivers of SW engagement and leadership buy-in, SW knowledge, skills, and comfort with LMSC, workflow changes for EMR documentation and distribution of safe storage devices, and ED interdisciplinary support. There was a significant increase in documentation for all types of lethal means after intervention 1. Despite a decrease in intervention 2 cycle rates for sharps/knives and medication documentation, firearm documentation continued to increase. This finding suggests a successful shift in prioritizing screening for firearm access, the leading cause of death for children.

During the past 5 years, many pediatric EDs have implemented interventions of varying reliability to improve LMSC documentation rates and counseling provided to families.^18,19,21^ One academic pediatric ED developed a documentation smart phrase for licensed clinical SWs and psychiatrists, as well as interventions for safe storage.^25^ Their published smart phrase included documentation on firearms, medications, sharps, barriers, and family agreement to safe storage measures and reached a rate of 80%.^25^ At a different academic pediatric ED, the SWs added a templated phrase about firearm access only in their consult notes, which included documentation of triple safe storage and whether a cable gun lock was distributed.^18^ Thirteen months following the start of this intervention, their documentation of firearm access increased from 37.8% to 81.6%.^18^ In a third study, an additional academic pediatric ED improved the documentation of firearms from 0% to more than 90% by adding standardized questions to ED behavioral health assessment templates and improving access to and restocking of cable gun locks.^19^ All 3 centers included questions and interventions for safe firearm storage. However, only the first ED mentioned included medications and sharps as part of its lethal means intervention. This project provides valuable information by comparing documentation rates among types of lethal means, as no centers have previously done so.

Unfortunately, evidence for creating operational measures to document elements for counseling on lethal means restrictions is limited,^27^ and no standardized documentation guidelines exist for the EMR.^28^ Verbiage and documentation practices vary among institutions.^29,30^ Some institutions include various forms of lethal means counseling that include medications, asphyxiation risk, alcohol, knives, chemical substances, and ligatures in their documentation, whereas others focus solely on firearms.^29,30^ A systematic review concluded that there is limited evidence on identifying the most effective methods to provide counseling to target populations, including those with mental health presentations.^29^ In response to this information gap, we modified documentation over time by creating a standardized PowerForm intended to serve as a model for other institutions for the comprehensive documentation of lethal means counseling.

This study focuses on the global aim of increasing LMSC documentation as a proxy for providing LMSC counseling to families. This is because counseling measures increase safe storage practices. A systematic review of 19 LMSC implementation studies found increases in behavioral changes, including improved rates of firearm storage practices, use of safe firearm storage devices (eg, cable gun locks), and reduced access to medications by locking or disposing of them.^30^ No studies were identified that examined behavior changes for other lethal means, including sharps.^30^ A pediatric-specific randomized control trial improved firearm storage by 100% and medication storage by 200% by counseling and providing free storage devices.^16^

### Firearm LMSC Documentation

This study demonstrated the highest rates of LMSC documentation for firearms. This is congruent with literature that demonstrates a higher focus on safe firearm storage counseling compared with medications, as well as increased adherence to safe storage practices. Of 19 studies examining changes in firearm storage practices after LMSC interventions, 13 reported significant improvement in firearm storage, 4 reported no statistical difference in change in behavior, and 2 did not test or report whether behavior changes were significant.^30^ Nine of 10 studies that assessed changes in the use of firearm locking devices (including cable gun locks) reported significant improvements in use.^30^ Youth with firearm access have 1.52 times higher odds of current suicidal ideation and 1.61 times higher odds of prior suicide attempt than youth without firearm access, demonstrating the importance of conducting LMSC for this patient population.^31^ Reaching a rate of 89% for firearm access documentation conveys the success of establishing our interventions to improve firearm safe storage documentation for adolescents with mental health concerns.

### Difference between Baseline and Intervention Groups

There was a significant decrease in the proportion of discharged patients in the intervention periods compared with the baseline period. This may have been due to the opening of our inpatient psychiatric facility in October 2022, which was associated with an increase in annual admissions. Additionally, a balancing measure for this study was that the median ED length of stay for admitted patients decreased. Therefore, our intervention did not negatively impact other patient care indicators.

Increased access and availability of inpatient psychiatric services for the greater catchment area may explain the higher proportion of admissions. This may be due to increased ED referrals and transfers with higher acuity, leading to higher admission rates across the catchment area. The opening and continuing expansion of inpatient psychiatric beds at our own facility were confounding factors that are difficult to account for. This decreases generalizability, as these changes are unlikely to hold in replication studies, where access to inpatient psychiatric beds is relatively stable by comparison.

### Limitations

This is a single-center study, and results with our interventions may not be generalizable to other centers. Additionally, the baseline period was limited to 4 months. However, LMSC documentation rates were significantly low, ranging from 1% to 3%, and anecdotally, our team had stated that earlier LMSC documentation rates were not any more consistent in previous periods.

All documentation was completed after the patient encounter, rather than in real time, which likely reduced adherence to and accuracy of responses collected retrospectively. Actual LMSC rates may have been higher in both the baseline and intervention periods if real-time documentation had been reviewed. Future work could explore real-time documentation using a tablet or ambient listening technology. Additionally, documentation was measured rather than counseling behavior or family behavior change during the implementation of home safe storage devices. EMR documentation is less accurate compared with observed patient-provider counseling interactions.

Finally, there was significant SW turnover during the intervention periods with many new hires, making standardization and onboarding a persistent challenge. These unacknowledged challenges may have contributed to barriers to improving documentation rates for medications and sharps.

## CONCLUSIONS

This quality improvement project demonstrates 2 successful interventions to improve ALD and CLD rates in a tertiary academic pediatric ED. Our multidisciplinary team implemented a novel documentation system for LMSC via an inclusive PowerForm and distributed secure storage devices. Through these actions, this study hopes to encourage efforts to distribute safe storage devices, analyze barriers to the safe storage of firearms, sharps, and medications, and enhance the practice and documentation of LMSC.

## ACKNOWLEDGMENTS

The authors thank Turaj Vazifendan for his contributions to the statistical analysis. They also thank the Children’s Hospital of the King’s Daughters ED SW team for their support.
